# Retrospective evaluation of novel serum inflammatory biomarkers in first-episode psychiatric disorders: diagnostic potential and immune dysregulation

**DOI:** 10.3389/fpsyt.2024.1442954

**Published:** 2024-12-11

**Authors:** Min Qiu, Chenkai Zhang, Haiqing Zhang, Hao Chen, Yingjia Lei, Ping Li, Shaochuan Zhang

**Affiliations:** ^1^ Department of Clinical Laboratory, The Mental Health Center of Kunming Medical University, Kunming, China; ^2^ Department of Clinical Laboratory, Yunnan Fuwai Cardiovascular Hospital, Kunming, China

**Keywords:** systemic inflammatory index, systemic inflammation response index, lipid metabolism, inflammatory biomarkers, psychiatric disorder

## Abstract

**Background:**

This study assessed the diagnostic capabilities of eight inflammatory biomarkers in first-episode schizophrenia (SCZ), bipolar disorder (BD), and depression (D), examining their differential expression across these psychiatric disorders. The markers studied include neutrophils/lymphocyte ratio (NLR), aggregate index of systemic inflammation (AISI), systemic immune-inflammation index (SII), systemic inflammation response index (SIRI), lymphocytes/high-density lipoprotein (HDL) ratio (LHR), monocytes/HDL ratio (MHR), neutrophils/HDL ratio (NHR), and platelets/HDL ratio (PHR).

**Methods:**

We conducted a retrospective observational study involving 335 individuals with SCZ, 68 with BD, 202 with D, and 282 healthy controls (C) to evaluate hematologic parameters from untreated patients and controls.

**Results:**

Significant differences in biomarker levels were found between patient groups and controls. Logistic regression analysis indicated that NHR and MHR (*p* < 0.001), as well as LHR and NLR (*p* < 0.01), were predictive factors for SCZ. MHR was a predictive factor for BD (*p* < 0.05). NHR (*p* < 0.01) and MHR (*p* < 0.001) were predictive factors for distinguishing between D and C. The area under the curve (AUC) value of the NHR + MHR + NLR composite index model for the SCZ group was 0.846 (*p* < 0.001). In the BD group, the AUC value for the MHR was 0.816 (*p* < 0.001). The D group’s combined AUC value of NHR + MHR was 0.824 (*p* < 0.001).

**Conclusion:**

This study highlights the diagnostic value of inflammatory biomarkers in distinguishing SCZ, BD, and D based on their differential expression.

## 1 Introduction

Psychiatric disorders, including schizophrenia (SCZ), bipolar disorder (BD), and depression (D), lack quantifiable biomarkers for diagnosis (1–3). These disorders affect approximately 10.7% of the global population, yet diagnostic processes remain subjective (4–7). Treatment is further complicated by severe adverse reactions and resistance to antipsychotic medications (8). Identifying objective biomarkers is crucial to improving diagnosis and understanding these conditions.

Recent research has explored underlying mechanisms of psychiatric disorders to better understand the pathophysiology and identify biomarkers, especially in early disease (9). Studying the neurological foundations of psychiatric disorders can facilitate the development of innovative treatments and improve diagnostic accuracy (10). Chronic low-level inflammation, characterized by a diminished yet ongoing reaction, is commonly observed in emotional, psychiatric, personality, and neurotic conditions (11). Elevated levels of pro-inflammatory cytokines, oxidative stress markers, chemokines, and soluble adhesion molecules have been observed with higher concentrations in the blood and cerebrospinal fluid of individuals with SCZ and BD (12). Similar associations have been reported with cytokines and neuropeptides in depression (13). Biomarker identification for diagnosing and monitoring treatment responses in psychiatric disorders is an essential research focus (9). Serum inflammatory biomarkers offer promising avenues for molecular diagnostics.

The pathogenesis of these illnesses has drawn increased attention to the role of inflammation in recent studies, suggesting a potential link between immune dysregulation and psychiatric symptoms. Specifically, serum inflammatory biomarkers are being explored to understand the causes of the early onset of psychiatric disorder and identify promising diagnostic candidates. Proposed hematological parameters include neutrophil-to-lymphocyte ratio (NLR), systemic immune-inflammation index (SII), systemic inflammation response index (SIRI), aggregate index of systemic inflammation (AISI), lymphocyte/high-density lipoprotein (HDL-C) ratio (LHR), monocytes/HDL-C ratio (MHR), neutrophils/HDL-C ratio (NHR), and platelet/HDL-C ratio (PHR) (14). These cost-effective parameters derived from routine complete blood counts and biochemical assays reflect inflammation and immune balance (15). Although inflammation’s role in mental health is better understood, the diagnostic potential of these serological markers in first-episode psychiatric disorders requires further exploration.

Several studies have examined single or combined indicators in psychiatric disorders (16–18). This study aims to evaluate whether these biomarkers can serve as predictive or early diagnostic tools for psychiatric disorders and enhance understanding of their pathophysiology. We hypothesize that biomarker levels will significantly differ between patients with first-episode psychiatric disorders and healthy controls, with some biomarkers acting as reliable predictors.

## 2 Materials and methods

### 2.1 Study design and participants

This retrospective cross-sectional study included patients with SCZ, BD, and D treated at the Mental Health Center of Kunming Medical University from January 2021 to December 2023.

#### 2.1.1 Setting

The study was conducted at the Mental Health Center of Kunming Medical University, which provides comprehensive mental health services.

#### 2.1.2 Participants

Patients diagnosed with SCZ, BD, and D based on ICD-10 criteria by psychiatry specialists were included. Inclusion criteria were (1): diagnosis of SCZ, BD, and D based on ICD-10 standards during the first visit and in the acute stage; (2) the presence of complete blood count parameters along with biochemical information; and (3) individuals aged 18 to 65 years at the moment of blood testing. Exclusion criteria were: (1) inflammatory or immunodeficiency disorders, systemic or localized inflammatory diseases, and (2) severe physical illness. Healthy controls (C) were aged between 18 and 65 during the blood analysis and had available biochemical and complete blood count data. Control participants were matched to experimental groups based on sample size.

#### 2.1.3 Variables

The primary variables were sociodemographic data and hematological parameters. Blood testing: Hematology specimens were tested using standard laboratory equipment (LABOSPECT 008AS, Sysmex XN 1000) following the laboratory’s standard operating procedures to ensure consistency and accuracy. Serum inflammatory biomarkers were calculated using the following formulas: NLR = Neutrophil Count (10^9^/L)/Lymphocyte Count (10^9^/L), SII = Platelet Count (10^9^/L) × Neutrophil Count (10^9^/L)/Lymphocyte Count (10^9^/L), SIRI = Neutrophil Count (10^9^/L) × Monocyte Count (10^9^/L)/Lymphocyte Count (10^9^/L), AISI = Platelet Count (10^9^/L) × Neutrophil Count (10^9^/L) × Monocyte Count (10^9^/L)/Lymphocyte Count (10^9^/L), LHR = Lymphocyte Count (10^9^/L)/HDL-C (mmol/L), MHR = Monocyte Count (10^9^/L)/HDL-C (mmol/L), NHR = Neutrophil Count (10^9^/L)/HDL-C (mmol/L), PHR = Platelet Count (10^9^/L)/HDL-C (mmol/L). Confounding factors, such as age and gender, were also considered.

#### 2.1.4 Data sources/measurement

Data were extracted from electronic medical records and the Laboratory Information System. Blood samples were collected between 6:00 and 7:30 AM under standardized protocols. Diagnostic data were cross-verified with ICD-10 criteria to minimize selection and information bias, and inclusion/exclusion criteria were strictly applied. Consistent sampling times and standardized diagnostic procedures ensured reliability.

### 2.2 Ethics statement

The low-risk retrospective observational study was approved by the Ethics Committee of the Mental Health Center of Kunming Medical University (approval number: YNJS-20230615-001). Informed consent was not required, and all data were anonymized to ensure confidentiality.

### 2.3 Statistical methods

Statistical analyses were conducted using SPSS 23.0. The Kolmogorov-Smirnov test was utilized to assess data normality. For non-normally distributed data and unequal variances, the Kruskal-Wallis test was applied, while one-way analysis of variance (ANOVA) was used for normally distributed data. *Post hoc* multiple comparisons were conducted using Bonferroni calibration. Descriptive statistics were analyzed with the chi-squared test, and continuous data are presented as mean ± standard deviation. Univariate analysis identified disease risk factors, with variables showing statistical significance (*p* < 0.05) included in multivariate logistic regression. Binary logistic regression was used to adjust for confounding variables such as gender and age. Receiver operating characteristic (ROC) curve analysis evaluated diagnostic factors, with the Youden index optimized to determine the ideal cutoff value. To minimize the influence of highly variable MHR values, MHR was scaled by multiplying by 10 (19). Combined measurement models constructed via binary logistic regression were assessed for their diagnostic application. A p-value of < 0.05 was considered statistically significant.

## 3 Results

### 3.1 Participant characteristics

Based on electronic medical records, the preliminary counts of eligible patients were 212 in the D group, 358 in the SCZ group, and 80 in the BD group. After excluding participants with incomplete data, the final analysis included 335 patients with SCZ, 68 with BD, 202 with D, and 282 as C. The SCZ group had an average age of 36.030 ± 13.43 years, consisting of 150 males and 185 females. The BD group had an average age of 34.882 ± 14.547 years, comprising 31 males and 37 females. The D group had an average age of 34.921 ± 13.772 years, comprising 70 male cases and 132 female cases. The C group, averaging 35.755 ± 7.172 years, comprised 108 males and 174 females. Statistical analysis showed no significant differences in age and sex between the patient and C groups (*p* > 0.05). There were no statistically significant age differences among the patient groups (*p* > 0.05). However, sex differences were statistically significant between the SCZ and D groups (*p* < 0.05), with a higher proportion of females in the D group (65%) compared to the SCZ group (55%). No statistically significant sex differences were observed among the other patient groups (*p* > 0.05).

### 3.2 Analysis of inflammatory biomarkers

In contrast to the C group, the SCZ group showed higher levels of NLR, NHR, LHR, MHR, PHR, AISI, SII, SIRI, white blood cell (WBC), neutrophils, and monocytes, while cholesterol (CHO), triglycerides (TG), HDL-C, and LDL-C were reduced (*p* < 0.05). Similarly, the BD group also exhibited increased levels of SII, SIRI, NHR, NLR, AISI, LHR, MHR, PHR, WBC, neutrophils, and monocytes, along with lower CHO, HDL-C, and LDL-C compared with the C-group (*p* < 0.05). The D group showed decreased CHO, TG, HDL-C, and LDL-C levels and elevated SIRI, NHR, LHR, MHR, PHR, AISI, WBC, and monocytes compared with the C group (*p* < 0.05).

**Figure 1:**
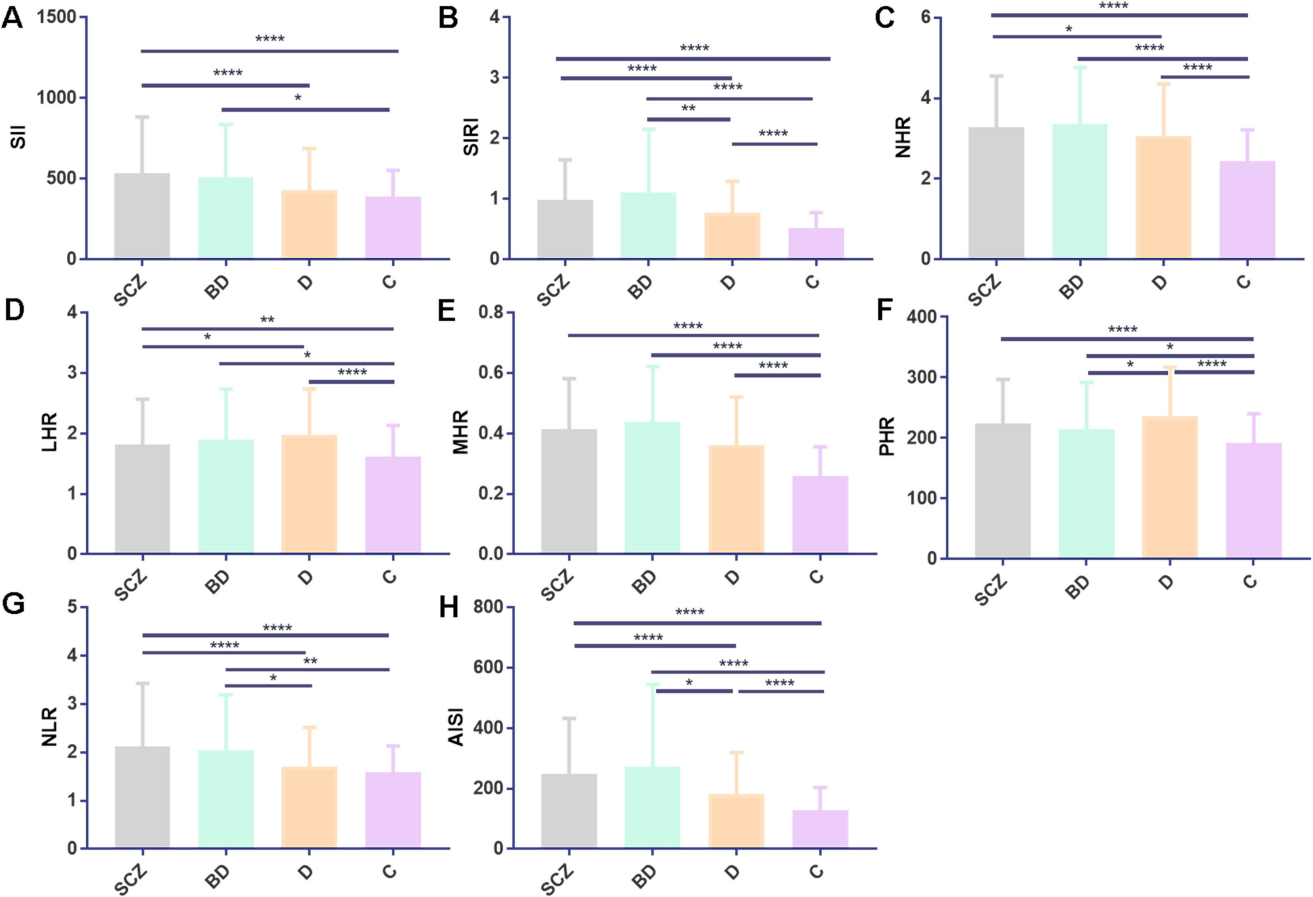
SCZ, BD, D, and C inflammatory ratios comparison. **(A–H)** depict comparisons for SII, SIRI, NHR, LHR, MHR, PHR, NLR, and AISI. Statistical significance is denoted as follows: *p < 0.05, **p < 0.01, and ****p < 0.0001. All values are presented as mean ± standard deviation. Abbreviations used include SCZ, schizophrenia; BD, bipolar disorder; D, depression; C, healthy control; SII, systemic immune-inflammation index; SIRI, systemic inflammation response index; NHR, neutrophil/HDL-C; LHR, lymphocyte/HDL-C; MHR, monocyte/HDL-C; PHR, platelet/HDL-C; NLR, neutrophils/lymphocytes; and AISI, aggregate index of systemic inflammation.

Comparing the indicators between the SCZ group and those in the BD group, no statistically significant difference was found (*p* > 0.05). SII, SIRI, NHR, NLR, AISI, WBC, and neutrophils were all higher in the SCZ group than in the D group, while LHR levels were lower in the D group (*p* < 0.05). The BD group had greater levels of SIRI, NLR, AISI, WBC, neutrophils, and monocytes than the D group, whereas the D group had lower PHR levels. Detailed results for all variables are summarized in **Table 1** and **Figure 1**.

**Table 1:** shows how the laboratory and demographic traits of healthy controls (C), bipolar disorder (BD), depression (D), and schizophrenia (SCZ) differ from one other.

Variables	SCZ (n = 335)	BD (n = 68)	D (n = 202)	C (n = 282)	*p*
Age (year)	36.030 ± 13.432	34.882 ± 14.547	34.921 ± 13.772	35.755 ± 7.172	0.108
Sex(male/female)	150/185	31/37	70/132^&^	108/174	0.082
SII (10^9^/mmol)	532.674 ± 350.386^*^	508.779 ± 328.512^*^	441.976 ± 278.923^&^	389.158 ± 163.741	<0.001
SIRI (10^9^/mmol)	0.981 ± 0.667^*^	1.102 ± 1.051^*^	0.771 ± 0.524^*&#^	0.525 ± 0.253	<0.001
NHR (10^9^/mmol)	3.278 ± 1.274^*^	3.356 ± 1.409^*^	3.060 ± 1.297^*&^	2.432 ± 0.784	<0.001
LHR (10^9^/mmol)	1.816 ± 0.754^*^	1.901 ± 0.839^*^	1.986 ± 0.755^*&^	1.614 ± 0.520	<0.001
MHR (10^9^/mmol)	0.414 ± 0.169^*^	0.439 ± 0.183^*^	0.408 ± 0.155^*^	0.261 ± 0.096	<0.001
PHR (10^9^/mmol)	223.771 ± 73.016^*^	214.111 ± 77.794^*^	235.455 ± 81.501^*#^	191.940 ± 47.761	<0.001
NLR	2.120 ± 1.311^*^	2.046 ± 1.153^*^	1.707 ± 0.877^&#^	1.584 ± 0.553	<0.001
AISI (10^18^/mmol)	249.672 ± 183.240^*^	273.506 ± 271.958^*^	200.019 ± 150.554^*&#^	129.915 ± 73.782	<0.001
WBC (10^9^/L)	6.413 ± 1.457^*^	6.602 ± 1.680^*^	6.087 ± 1.331^*&#^	5.597 ± 1.110	<0.001
Neutrophil (10^9^/L)	3.746 ± 1.284^*^	3.837 ± 1.279^*^	3.314 ± 1.190^&#^	3.084 ± 0.784	<0.001
Lymphocyte (10^9^/L)	2.037 ± 0.675	2.167 ± 0.747	2.151 ± 0.649	2.043 ± 0.487	0.236
Monocyte (10^9^/L)	0.465 ± 0.148^*^	0.505 ± 0.192^*^	0.439 ± 0.125^*#^	0.330 ± 0.098	<0.001
Platelet (10^9^/L)	252.761 ± 58.130	246.235 ± 61.750	256.757 ± 67.033	244.823 ± 43.261	0.423
CHO (mmol/L)	4.226 ± 0.931^*^	4.346 ± 1.176^*^	4.105 ± 0.880^*^	4.805 ± 0.870	<0.001
TG (mmol/L)	1.295 ± 0.876^*^	1.384 ± 0.752	1.309 ± 0.846^*^	1.369 ± 0.660	0.021
HDL-C (mmol/L)	1.194 ± 0.328^*^	1.209 ± 0.280^*^	1.136 ± 0.243^*^	1.318 ± 0.261	<0.001
LDL-C (mmol/L)	2.385 ± 0.691^*^	2.523 ± 0.811^*^	2.346 ± 0.677^*^	2.816 ± 0.707	<0.001

SCZ, schizophrenia; BD, bipolar disorder; D, depression; C, healthy control; SII, systemic immune-inflammation index; SIRI, systemic inflammatory response index; NHR, neutrophil/HDL-C; LHR, lymphocyte/HDL-C; MHR, monocyte/HDL-C; PHR, platelet/HDL-C; NLR, neutrophils/lymphocytes; AISI: total systemic inflammation index; WBC: white blood cells; CHO, cholesterol; TG, triglycerides; HDL-C, high-density lipoprotein cholesterol; LDL-C, and low-density lipoprotein cholesterol. ^*^ vs. C group, *p* < 0.05; ^&^ vs. SCZ group, *p* < 0.05; ^#^ vs. BD group, *p* < 0.05.

### 3.3 Comparison of inflammatory biomarkers between bipolar manic and bipolar depressive episodes

The BD group included 36 individuals with bipolar depressive episodes (BD-D) and 22 individuals with bipolar manic episodes (BD-M). No significant age or gender differences were observed between patient groups and controls, nor within patient subgroups. When comparing the BD-D group with the D group, the BD-D group exhibited higher triglyceride (TG) levels and lower PHR levels (*p* < 0.05). Detailed findings are presented in **Table 2**.

**Table 2:** Comparison of demographic and laboratory characteristics between bipolar disorder depressive episodes (BD-D), bipolar disorder manic episodes (BD-M), depression (D), and healthy control group (C).

Variables	BD-D (n = 36)	D (n = 202)	BD-M (n = 22)	C (n = 282)	*p*
Age (year)	35.917 ± 14.645	34.921 ± 13.772	37.591 ± 15.909	35.755 ± 7.172	0.338
Sex (male/female)	17/19	70/132	10/12	108/174	0.426
SII (10^9^/mmol)	441.839 ± 306.271^	441.976 ± 278.923^	644.692 ± 378.993^*^	389.158 ± 163.741	0.005
SIRI (10^9^/mmol)	0.972 ± 1.142^*^	0.771 ± 0.524^*^^	1.459 ± 1.046^*^	0.525 ± 0.253	<0.001
NHR (10^9^/mmol)	3.066 ± 1.227^*^^	3.060 ± 1.297^*^	3.898 ± 1.590^*^	2.432 ± 0.784	<0.001
LHR (10^9^/mmol)	2.006 ± 0.981	1.986 ± 0.755^*^	1.753 ± 0.702	1.614 ± 0.520	<0.001
MHR (10^9^/mmol)	0.427 ± 0.209^*^	0.408 ± 0.155^*^	0.472 ± 0.166^*^	0.261 ± 0.096	<0.001
PHR (10^9^/mmol)	209.969 ± 92.734^&^	235.455 ± 81.501^*^	211.169 ± 58.466	191.940 ± 47.761	<0.001
NLR	1.807 ± 0.978	1.707 ± 0.877^	2.592 ± 1.431^*^	1.584 ± 0.553	0.006
AISI (10^18^/mmol)	238.998 ± 298.31^*^	200.019 ± 150.554^*^^	360.091 ± 266.811^*^	129.915 ± 73.782	<0.001
WBC (10^9^/L)	6.359 ± 1.812^*^	6.087 ± 1.331^*^^	7.215 ± 1.360^*^	5.597 ± 1.110	<0.001
Neutrophil (10^9^/L)	3.552 ± 1.055	3.314 ± 1.190^	4.474 ± 1.445^*^	3.084 ± 0.784	<0.001
Lymphocyte (10^9^/L)	2.287 ± 0.840	2.151 ± 0.649	2.028 ± 0.691	2.043 ± 0.487	0.249
Monocyte (10^9^/L)	0.499 ± 0.239^*^	0.439 ± 0.125^*^^	0.539 ± 0.129^*^	0.330 ± 0.098	<0.001
Platelet (10^9^/L)	241.889 ± 67.974	256.757 ± 67.033	247.682 ± 63.662	244.823 ± 43.261	0.449
CHO (mmol/L)	4.388 ± 1.073^*^	4.105 ± 0.880^*^	4.303 ± 1.454	4.805 ± 0.870	<0.001
TG (mmol/L)	1.453 ± 0.675^&^	1.309 ± 0.846^*^	1.378 ± 0.904	1.369 ± 0.660	0.061
HDL-C (mmol/L)	1.230 ± 0.306	1.136 ± 0.243^*^	1.207 ± 0.270	1.318 ± 0.261	<0.001
LDL-C (mmol/L)	2.443 ± 0.753^*^	2.346 ± 0.677^*^	2.612 ± 0.938	2.816 ± 0.707	<0.001

BD-D, bipolar disorder depressive episode; BD-M, bipolar disorder manic episode; D, depression; C, healthy control; SII, systemic immune-inflammation index; SIRI, systemic inflammatory response index; NHR, neutrophil/HDL-C; LHR, lymphocyte/HDL-C; MHR, monocyte/HDL-C; PHR, platelet/HDL-C; NLR, neutrophils/Lymphocytes; AISI, aggregate index of systemic inflammation; WBC, white blood cells; CHO, cholesterol; TG, triglycerides; HDL-C, high-density lipoprotein cholesterol; and LDL-C, low-density lipoprotein cholesterol. ^*^ vs. C group, *p* < 0.05; ^&^ vs. D group, *p* < 0.05; and ^ vs BD-M group, *p* < 0.05.

### 3.4 The contributing factors of SCZ, BD, and D incidence were analyzed using both single-factor and multivariate logistic regression

Significant variables from univariate logistic regression were included in the multivariate model to mitigate confounding effects to derive adjusted OR. The multivariate analysis identified independent associations between NHR, LHR, MHR, and NLR. Specifically, LHR, MHR, and NLR positively associated with SCZ, while NHR showed a negative association. Moreover, MHR exhibited an independent positive association with BD. Regarding D, NHR, MHR, and sex were identified as independent risk factors, with MHR showing a positive association and NHR showing a negative association. Detailed results are presented in **Table 3**.

**Table 3:** Results of SCZ, B, and D binary logistic regression analysis.

Variables	SCZ (n = 335)			BD (n = 68)			D (n = 202)		
	OR	95% CI	*p*	OR	95% CI	*p*	OR	95% CI	*p*
SII	0.996	0.988-1.003	0.273	1.005	0.990-1.021	0.503	1.001	0.991-1.011	0.906
SIRI	0.520	0.002-108.225	0.810	73.531	0.008-682126	0.356	3.181	0.008-1335	0.707
NHR	0.284	0.165-0.489	<0.001	0.895	0.432-1.854	0.766	0.379	0.208-0.692	0.002
LHR	4.192	1.704-10.311	0.002	1.543	0.448-5.311	0.492	2.236	0.869-5.749	0.095
MHR*10	2.727	1.666-4.464	<0.001	2.731	1.235-6.042	0.013	3.139	1.828-5.393	<0.001
PHR	1.003	0.995-1.011	0.456	0.993	0.979-1.007	0.298	1.005	0.996-1.013	0.273
NLR	17.351	2.221-135.513	0.007	0.340	0.006-17.903	0.594	2.191	0.139-34.452	0.577
AISI	1.007	0.988-1.027	0.454	0.988	0.955-1.023	0.503	0.997	0.976-1.019	0.806
Age	1.011	0.991-1.031	0.281	0.987	0.952-1.023	0.467	1.008	0.985-1.031	0.497
Sex	1.392	0.905-2.143	0.133	1.770	0.853-3.674	0.125	2.361	1.433-3.889	0.001
Hosmer–Lemeshow goodness-of-fit test		6.548 (*p* = 0.586)		12.829 (*p* = 0.118)			7.439 (*p* = 0.490)		

SCZ, schizophrenia; BD, bipolar disorder; D, depression; SII, systemic immune-inflammation index; SIRI, systemic inflammatory response index; NHR, neutrophil/HDL-C; LHR, lymphocyte/HDL-C; MHR, monocyte/HDL-C; PHR, platelet/HDL-C; NLR, neutrophils/lymphocytes; and AISI, aggregate index of systemic inflammation.

### 3.5 ROC curve analysis

**Figure 2:**
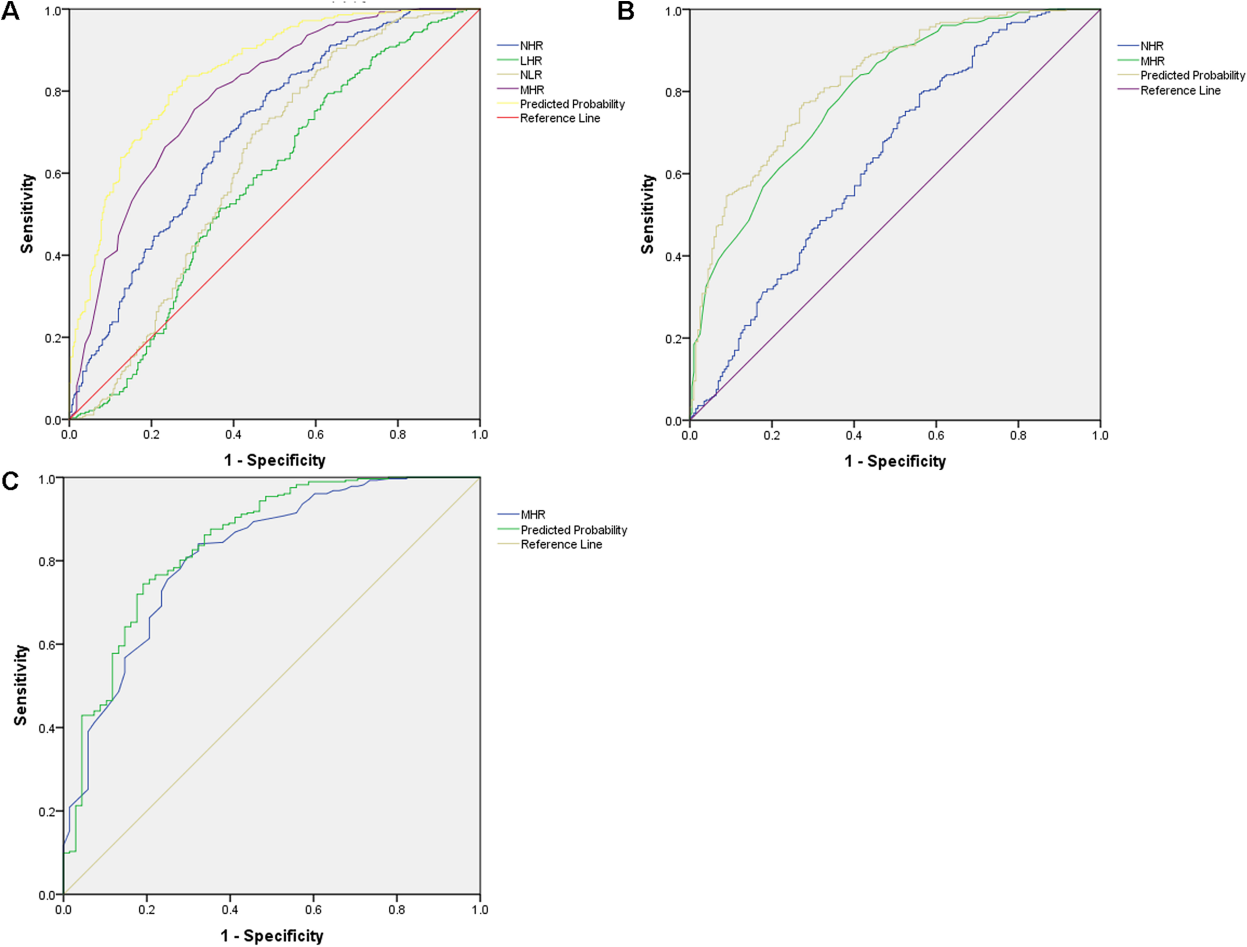
ROC curve analysis of inflammatory markers in predicting psychiatric disorder. **(A)** ROC curve for SCZ, NHR [AUC 0.703 (0.662–0.743), *p* < 0.001, critical value 2.831]; MHR [AUC 0.790 (0.755–0.826), *p* < 0.001, critical value 3.15]; NLR [AUC 0.625 (0.581–0.669), *p* < 0.001, critical value 2.123]; the AUCs of LHR are lower than 0.6. Indicator combination model (NHR + MHR + NLR) [AUC 0.846 (0.816–0.876), *p* < 0.001, critical value 0.550]. **(B)** ROC curve for D, NHR [AUC 0.638 (0.586–0.689), *p* < 0.001, critical value 2.984]; MHR [AUC 0.795 (0.755–0.834), *p* < 0.001, critical value 3.450]. Indicator combination model (NHR + MHR) [AUC 0.824 (0.787–0.861), *p* < 0.001, critical value 0.398]. **(C)** ROC curve for BD, MHR [AUC 0.816 (0.758–0.875), *p* < 0.001, critical value 3.550].

In SCZ, NHR [area under the curve (AUC) 0.703 (0.662–0.743), *p* < 0.001, critical value 2.831, sensitivity 74.5%, specificity 57.6%] and MHR [AUC 0.790 (0.755–0.826), *p* < 0.001, critical value 3.15, sensitivity 75.5%, specificity 69.6%] demonstrated AUC values > 0.6, as indicated by the ROC curve. NLR showed AUC 0.625 (0.581–0.669), *p* < 0.001, critical value 2.123, sensitivity 89.7%, and specificity 35.8%. However, LHR had AUC values < 0.6. The diagnostic efficiency was enhanced by the indicator combination model (NHR + MHR + NLR), showing an AUC value of 0.846 (0.816–0.876), with statistical significance (*p* < 0.001), a critical value of 0.550, a sensitivity of 82.6%, and a specificity of 72.5%. The data are presented in **Figure 2A**.

For D, NHR showed an AUC of 0.638 (0.586–0.689), with statistical significance (*p* < 0.001), critical value 2.984, sensitivity 79.4%, and specificity 44.1%. MHR showed a higher AUC of 0.795 (0.755–0.834), with statistical significance (*p* < 0.001), a critical value of 3.450, a sensitivity of 82.3%, and a specificity of 60.4%. The combined model (NHR + MHR) enhanced diagnostic parameters, yielding an AUC of 0.824 (0.787–0.861), with statistical significance (*p* < 0.001), a critical value of 0.398, a sensitivity of 77.3%, and a specificity of 72.3%. These findings are illustrated in **Figure 2B**.

For BD, MHR achieved an AUC of 0.816 (0.758–0.875), with statistical significance (*p* < 0.001), a critical value of 3.550, a sensitivity of 84%, and a specificity of 67.6%. The data are presented in **Figure 2C**.

## 4 Discussion

Recent studies have identified SII, SIRI, AISI, NLR, LHR, MHR, NHR, and PHR as promising inflammation biomarkers. These markers are accessible, cost-effective, and clinically valuable (20, 21). However, their relationship with major first-episode psychiatric disorders remains unexplored. To the best of our knowledge, few studies have examined the association between these biomarkers and first-episode psychiatric disorders. Our retrospective cross-sectional study highlights significant alterations in these biomarkers among patients such patients, suggesting their potential diagnostic utility.

When compared with the C group, there were significant differences in multiple indicators in the SCZ group, BD group, and D group. The SCZ group had higher SII, SIRI, NHR, LHR, MHR, PHR, NLR, and AISI levels than the C group. The BD group also showed higher levels of these biomarkers than the C group. The D group also demonstrated higher SIRI, NHR, LHR, MHR, PHR, and AISI levels than the C group. These findings suggest a generalized inflammatory response across patient populations with first-episode psychiatric disorders, which may contribute to disease onset and progression.

Multivariate logistic regression analysis revealed that NHR, LHR, MHR, and NLR were independently associated with SCZ. MHR was found to be independently associated with BD, while NHR, MHR, and sex were identified as independent risk factors for D. Specifically, MHR, NHR, and NLR effectively distinguished SCZ from C, with MHR alone differentiating BD from C. For D, NHR and MHR served as reliable predictors. Notably, combined indicator models outperformed single-indicator models, with higher AUC values, underscoring the superior predictive power of combined biomarker assessments.

These findings have important clinical implications. Elevated inflammatory biomarkers may guide the development of targeted anti-inflammatory therapies and provide a foundation for personalized treatment strategies. Monitoring these biomarkers could also aid in evaluating therapeutic efficacy and tracking disease progression, offering a more dynamic approach to managing first-episode psychiatric disorders.

When directly compared, no significant statistical differences were noted between the BD and SCZ cohorts. However, the SCZ group displayed elevated levels of several inflammatory biomarkers, including SII, SIRI, NHR, NLR, AISI, WBC, and neutrophils, compared to the D group. In contrast, the SCZ group had lower LHR levels. Similarly, the BD group exhibited higher levels of SIRI, NLR, AISI, white blood cells, neutrophils, and monocytes but lower levels of PHR than the D group. Subgroup analysis within the BD cohort revealed that patients in the BD-D group had higher TG levels and lower PHR levels than the D group. These findings underscore differences in biomarkers and lipid metabolism among patient groups, providing insights into the heterogeneity of biological characteristics and disease manifestations.

NHR and MHR represent key indicators of the interplay between systemic inflammation and lipid metabolism. NHR reflects the dynamic relationship between neutrophils and HDL-C. Neutrophils, when activated, produce oxidants through enzymatic activity, promoting the oxidation of HDL-C and impairing its CHO efflux capability (22). Conversely, HDL-C suppresses neutrophil activation, adhesion, proliferation, and migration (23). Mendelian randomization studies have established a positive association between SCZ and leukocyte counts, including neutrophils (OR 1.013, *p* = 0.004) and monocytes (OR 1.018, *p* = 4.60 × 10^-4^) (24). Additionally, *post-hoc* analyses have reported elevated immature neutrophil levels in SCZ and BD, positively correlating with TG and inversely correlating with HDL-C (25). MHR signifies the interaction between monocytes and HDL-C levels, reflecting immune and metabolic interactions. Notably, significant differences in immune-related gene expression within monocytes have been documented in SCZ patients, implicating cell heterogeneity and immune dysregulation in the disease pathogenesis (26). Consistent with findings by Zhu et al. (27), in a Chinese cohort, our study observed significantly elevated NLR levels in patients with SCZ, reinforcing the inflammation hypothesis of SCZ. Similarly, Wei et al. (21, 28) demonstrated that MHR was significantly elevated in patients with BD compared to controls. In contrast, MHR was elevated in patients with D. These findings suggest that NHR, MHR, and NLR hold diagnostic promise in distinguishing and understanding the pathophysiological mechanisms of first-episode psychiatric disorders.

We identified dyslipidemia as a significant factor associated with psychiatric disorders. Specifically, we observed that patients with psychiatric disorders such as SCZ, BD, and D had abnormal levels of blood lipid indicators and lower blood lipid levels. SCZ and D patients had lower CHO, TG, HDL-C, and LDL-C levels, while patients with BD showed lower levels of CHO, HDL-C, and LDL-C. These alterations may result from common features of psychiatric disorders, such as malnutrition, anorexia, and metabolic dysregulation. Dyslipidemia may also affect nervous system function through neurotransmitter synthesis and release pathways, nerve cell membrane stability, and neuroinflammatory responses. Lipids are integral to cellular processes, including neurotransmission, neuroplasticity, neural projection myelination, energy metabolism, and neuroinflammation (29). Serum lipid profiles have also been associated with cognitive functioning in patients with SCZ, with TC and TG levels negatively correlated with cognitive scores. Serum lipid profiles may, therefore, serve as indicators for evaluating SCZ symptoms (30, 31). Beyond blood lipid abnormalities, disruptions in other lipid functions have been implicated in SCZ pathophysiology (32). In major depressive disorder, the MANF/EWSR1/ANXA6 pathway has been suggested as a potential link between hypolipidemia and major depression (33). Dyslipidemia in psychiatric disorders is further complicated by the effects of pharmacologic treatments, such as antipsychotics and antidepressants, which can alter lipid metabolism (34–36). These findings emphasize the need for mechanistic studies exploring lipid dysregulation’s role in psychiatric disorders and evaluating whether lipid-level adjustments could aid in preventing and treating these conditions.

However, this study has limitations. As a single-center retrospective analysis, the sample size may not be sufficient to establish definitive causal associations between identified biomarkers and psychiatric pathophysiology. This limitation introduces potential selection bias, restricting the generalizability of the findings. Although our study revealed strong associations between biomarkers and first-episode psychiatric disorders, further research is needed to validate these markers as distinct risk factors. Additionally, the study lacks long-term follow-up data and dynamic monitoring of inflammatory biomarkers. Consequently, it remains uncertain whether changes in these markers correlate with disease severity and progression. Future prospective studies with larger, more diverse sample sizes and longitudinal designs are critical to addressing these limitations. Such investigations could further elucidate the clinical utility of these biomarkers in diagnosing and managing first-episode psychiatric disorders and assess the therapeutic potential of targeting lipid and inflammatory dysregulation.

In conclusion, immune-inflammatory reactions and dyslipidemia are significant factors in the development of psychiatric disorders such as SCZ, BD, and D. These findings enhance our understanding of these conditions and offer insights for future prevention and treatment strategies. Further investigation is required to validate these findings, explore potential molecular pathways, and thoroughly evaluate their therapeutic application.

## Data Availability

The raw data supporting the conclusions of this article will be made available by the authors, without undue reservation.
